# The development of a conceptual framework on PrEP stigma among adolescent girls and young women in sub‐Saharan Africa

**DOI:** 10.1002/jia2.26213

**Published:** 2024-02-20

**Authors:** Miriam Hartmann, Laura Nyblade, Sophie Otticha, Tozoe Marton, Kawango Agot, Sarah T. Roberts

**Affiliations:** ^1^ Women's Global Health Imperative RTI International Berkeley California USA; ^2^ Department of Global Public Health Karolinska Institutet Stockholm Sweden; ^3^ Impact Research Development Organization Kisumu Kenya

**Keywords:** pre‐exposure prophylaxis, adolescent girls and young women, Kenya, stigma, conceptual framework, sexual and reproductive health

## Abstract

**Introduction:**

Stigma is a well‐known barrier to HIV testing and treatment and is an emerging barrier to pre‐exposure prophylaxis (PrEP) use. To guide future research, measurement and interventions, we developed a conceptual framework for PrEP stigma among adolescent girls and young women (AGYW) in sub‐Saharan Africa, a priority population for PrEP.

**Methods:**

A literature review, expert consultations and focus group discussions (FGDs) were conducted to adapt the Health Stigma and Discrimination Framework, describing the stigmatization process nested within the socio‐ecological framework. We reviewed all articles on PrEP stigma and on HIV, contraceptive or sexuality stigma among AGYW from 2009 to 2019. Expert consultations were conducted with 10 stigma or PrEP researchers and two Kenyan youth advisory boards to revise the framework. Finally, FGDs were conducted with AGYW PrEP users (4 FGDs; *n* = 20) and key influencers (14 FGDs; *n* = 72) in Kenya with the help of a Youth Research Team who aided in FGD conduct and results interpretation. Results from each phase were reviewed and the framework was updated to incorporate new and divergent findings. This was validated against an updated literature search from 2020 to 2023.

**Results:**

The conceptual framework identifies potential drivers, facilitators and manifestations of PrEP stigma, its outcomes and health impacts, and relevant intersecting stigmas. The main findings include: (1) PrEP stigma is driven by HIV, gender and sexuality stigmas, and low PrEP community awareness. (2) Stigma is facilitated by factors at multiple levels: policy (e.g. targeting of PrEP to high‐risk populations), health systems (e.g. youth‐friendly service availability), community (e.g. social capital) and individual (e.g. empowerment). (3) Similar to other stigmas, manifestations include labelling, violence and shame. (4) PrEP stigma results in decreased access to and acceptability of PrEP, limited social support and community resistance, which can impact mental health and decrease PrEP uptake and adherence. (5) Stigma may engender resilience by motivating AGYW to think of PrEP as an exercise in personal agency.

**Conclusions:**

Our PrEP stigma conceptual framework highlights potential intervention targets at multiple levels in the stigmatization process. Its adoption would enable researchers to develop standardized measures and compare stigma across timepoints and populations as well as design and evaluate interventions.

## 1 INTRODUCTION

Adolescent girls and young women (AGYW, ages 15–24) in sub‐Saharan Africa are highly vulnerable to HIV, accounting for 25% of new infections in this region despite composing just 10% of the population [1]. Although oral HIV pre‐exposure prophylaxis (PrEP) has the potential to reduce this burden, uptake and continuation rates have been low. In the 2016–2017 SEARCH study in Kenya and Uganda, only 19% of AGYW at elevated risk of HIV initiated PrEP [2]. Across the region, persistence has averaged 2 months or less [3, 4, 5], and only 5%–50% of PrEP users have high adherence after 3 months of use [5, 6, 7, 8]. Strategies to increase effective PrEP use are urgently needed to maximize its HIV prevention benefit among AGYW.

Stigma is a well‐known barrier to HIV testing and treatment [9, 10, 11] and is emerging as a central barrier to PrEP use [12, 13, 14, 15, 16]. Because adolescence is a critical time for identity formation and heightened sensitivity to others’ opinions [17], AGYW may be especially vulnerable to PrEP stigma and its consequences. In qualitative studies, AGYW using PrEP in Kenya, Tanzania and South Africa have expressed fears about being perceived as HIV positive or promiscuous [18, 19, 20, 21, 22, 23, 24, 25, 26]. They have reported anticipated and enacted stigma from peers, sexual partners, family members, healthcare providers (HCPs) and community members, and described stigma as a barrier to PrEP uptake, continuation and disclosure. Quantitative studies have also begun to measure the burden of PrEP stigma among AGYW in sub‐Saharan Africa and the United States [27, 28, 29], though none have reported associations with PrEP outcomes to date. In quantitative studies conducted among men who have sex with men (MSM) globally and adult women in the United States, PrEP stigma was a significant barrier to uptake and use [30, 31, 32, 33, 34, 35, 36].

Despite this growing body of research, there are currently, to our knowledge, no conceptual models of PrEP stigma to guide intervention development. A model of how PrEP stigma develops, and the mechanisms through which it influences health‐related outcomes, could play a critical role in identifying intervention targets to reduce PrEP stigma and mitigate its consequences, designing interventions and policies to address those targets, and ensuring rigorous evaluation of stigma‐reduction interventions. Conceptual models could facilitate more rapid growth of the knowledge base in this nascent field, by identifying areas where findings reinforce, complement or contrast each another, and where gaps remain. Recognizing that drivers, experiences and consequences of stigma may be context‐specific, we sought to develop a conceptual framework for PrEP stigma among AGYW in sub‐Saharan Africa to guide future research, measurement and interventions. Our framework was adapted from the Health Stigma and Discrimination Framework (HSDF) [37], which describes five domains of stigma operating across multiple levels of the socio‐ecological framework: (1) drivers (i.e. main negative factors driving stigma); (2) facilitators (i.e. positive or negative influences that exacerbate or mitigate stigma); (3) intersecting stigmas (i.e. when individuals are “marked” by stigmas associated with different identities); (4) stigma manifestations (i.e. experiences and practices); (5) outcomes and impacts. We chose this framework over existing HIV stigma frameworks, which also include influence across socio‐ecological levels and multiple domains of stigma, because it comprehensively includes levels of influence, stigma domains, as well as stigma within the healthcare sector alongside community‐based stigma because these are critical sources of stigma for AGYW. This gap was identified in a recent review of HIV stigma frameworks [38].

## 2 METHODS

**Figure 1:**
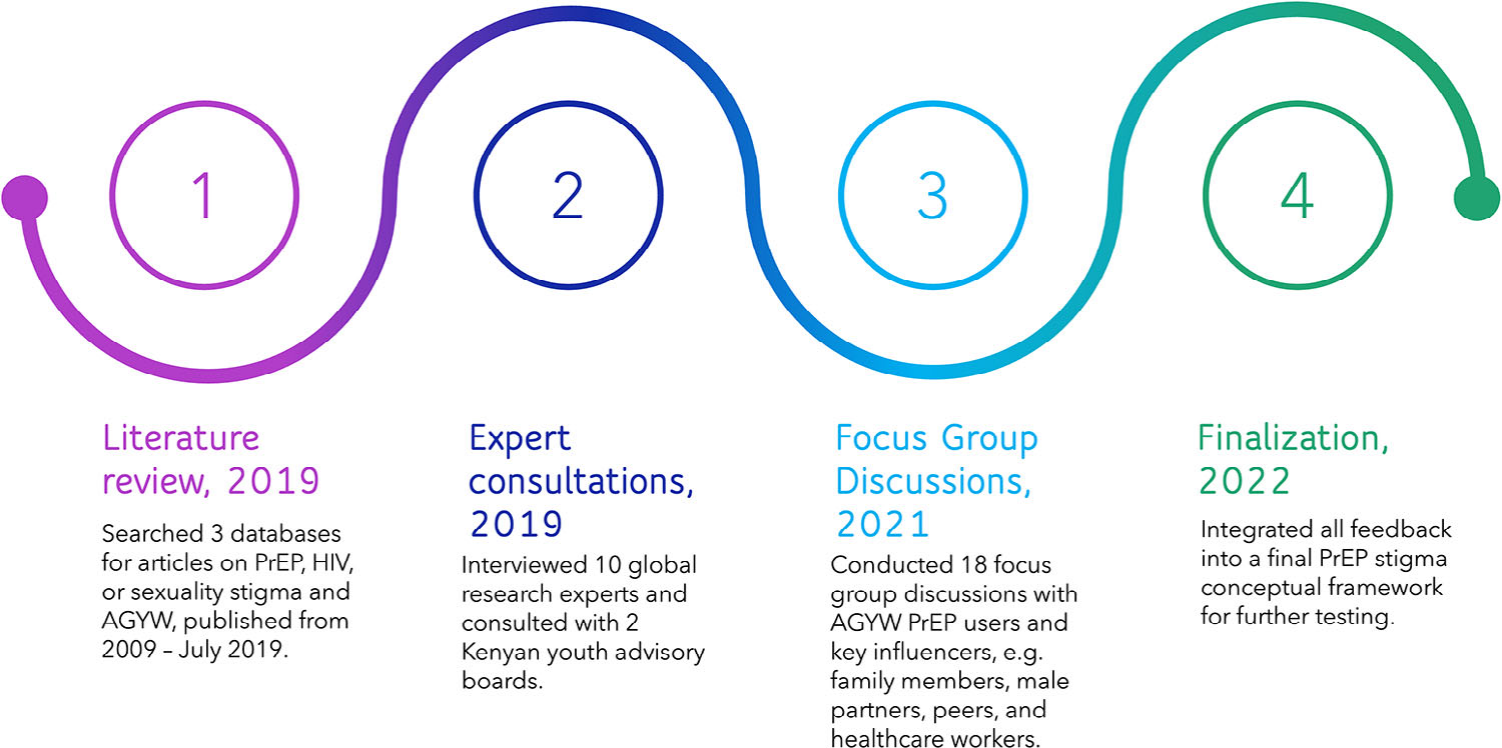
AGYW PrEP stigma conceptual framework development roadmap. Abbreviations: ARV, antiretroviral drug; HIV, human immunodeficiency virus; PrEP, pre‐exposure prophylaxis; SRH, sexual reproductive health; STIs, sexually transmitted infections.

We adapted the HSDF through an initial three‐phase process: a literature review, expert consultations and focus group discussions (FGDs) with AGYW and their key influencers (Figure 1). In each phase, data and feedback were analysed through the lens of the framework and used to populate and refine domains. Lastly, we updated the initial literature review to examine the final framework for continued relevance in light of the most recent PrEP literature published while the framework was being developed.

### 2.1 Phase 1: Literature review

PubMed, Web of Science and PsychINFO were searched for articles published in English from January 2009 to July 2019 on the topics of stigma or discrimination; AGYW; and PrEP, contraception, HIV or sexuality (see Supplementary Materials for search terms). From 712 articles identified, duplicates were removed and titles and abstracts of remaining articles were reviewed to identify those related to PrEP stigma (among any population) or HIV, contraceptive or sexuality stigma among AGYW. This resulted in a full review of 45 research articles, 2 commentaries, 5 reviews and 5 conference proceedings. Articles were abstracted using Excel to document the article type, study location, population and sample size, and content related to framework domains (e.g. drivers, facilitators, intersecting stigmas, etc.). Summary memos were developed for each domain, and then discussed among the research team, comprised of researchers from the United States and Kenya with experience in AGYW, stigma and PrEP, to draft the initial framework.

### 2.2 Phase 2: Expert consultations

Individual and group consultations with key topic experts were conducted to review framework findings. Individual interviews were conducted with willing researchers worldwide (*n* = 10: 6 PrEP experts, 4 stigma experts) identified based on the team's knowledge of the field and recommendations from experts. A total of 15 experts were contacted. Seven of the 10 interviewed were based in sub‐Saharan Africa. They were presented with the draft framework and findings by domain. We sought feedback on resonance and identification of gaps. Interviews were primarily phone‐based, with two in‐person, and were recorded for note‐taking. Notes were summarized into analysis memos highlighting themes by domain, and findings integrated into the framework. Two group consultations were conducted with youth advisory boards (YAB) in Kenya, from one organization in Kisumu (*n* = 4) and one in Nairobi (*n* = 12) to represent urban and peri‐urban settings. Consultations utilized an activity‐based approach to discuss PrEP stigma, including what it is, how, where and why it manifests, its impact, and resilience to stigma among youth. Findings were incorporated into the framework, which was reviewed by the research team and a youth research team (YRT) of eight AGYW aged 18–24 and revised through discussion.

### 2.3 Phase 3: FGDs with AGYW and key influencers

The final phase included 18 FGDs with AGYW PrEP users (4 FGDs; *n* = 20) and key influencers (14 FGDs; *n* = 72) in Siaya County, Kenya, where our parent research study was based. PrEP users included AGYW who accessed PrEP in the past 6 months through the Ministry of Health (MoH) or the DREAMS Initiative [39]. Influencers included peers (4 FGDs; *n* = 22), family members (4 FGDs; *n* = 23), male partners (3 FGDs; *n* = 12) and HCPs (3 FGDs; *n* = 15). “Peers” were AGYW who had never used PrEP, recruited from the same facilities and DREAMS sites as AGYW PrEP users. Family members and male partners were recruited based on recommendations from AGYW participants. HCPs were recruited from DREAMS, MoH and other PrEP delivery programmes. All FGDs were conducted by trained research assistants and YRT members in Dholuo or English with note‐takers. Facilitators used semi‐structured guides to explore perspectives on what contributes to PrEP stigma, who perpetrates it and how, and potential stigma‐reduction interventions. Immediately following each FGD, facilitators completed debriefing reports, summarizing key themes. All FGDs were audio‐recorded and transcribed in English.

Two levels of analysis were conducted. FGDs with PrEP users were completed first, and debriefing reports were rapidly analysed to refine the framework and inform the development of a measurement tool [40]. This analysis was completed through a participatory workshop with the research team, including YRT. During the workshop, the team reviewed debriefing reports and visually matched points to framework domains using sticky notes.

The second level of analysis included transcript coding. An initial codebook was developed by a study co‐investigator and reviewed by the research team. Changes were integrated and tested through application to a transcript by a six‐member coding team (four Kenyan, two US). Throughout coding, intercoder reliability was maintained through exercises with 20% of transcripts, where coders would code the same transcript, discuss discrepancies and find consensus, refining the codebook as needed. The team analysed code reports for each domain and developed analysis memos summarizing themes, noting differences and similarities by participant group, which were integrated into the framework.

The study was approved by the Maseno University Ethics Review Committee in Kenya. All participants provided informed consent, or parental consent and assent, prior to participation. FGD participants received compensation for their time and travel (∼$3.50).

## 3 RESULTS

**Figure 2:**
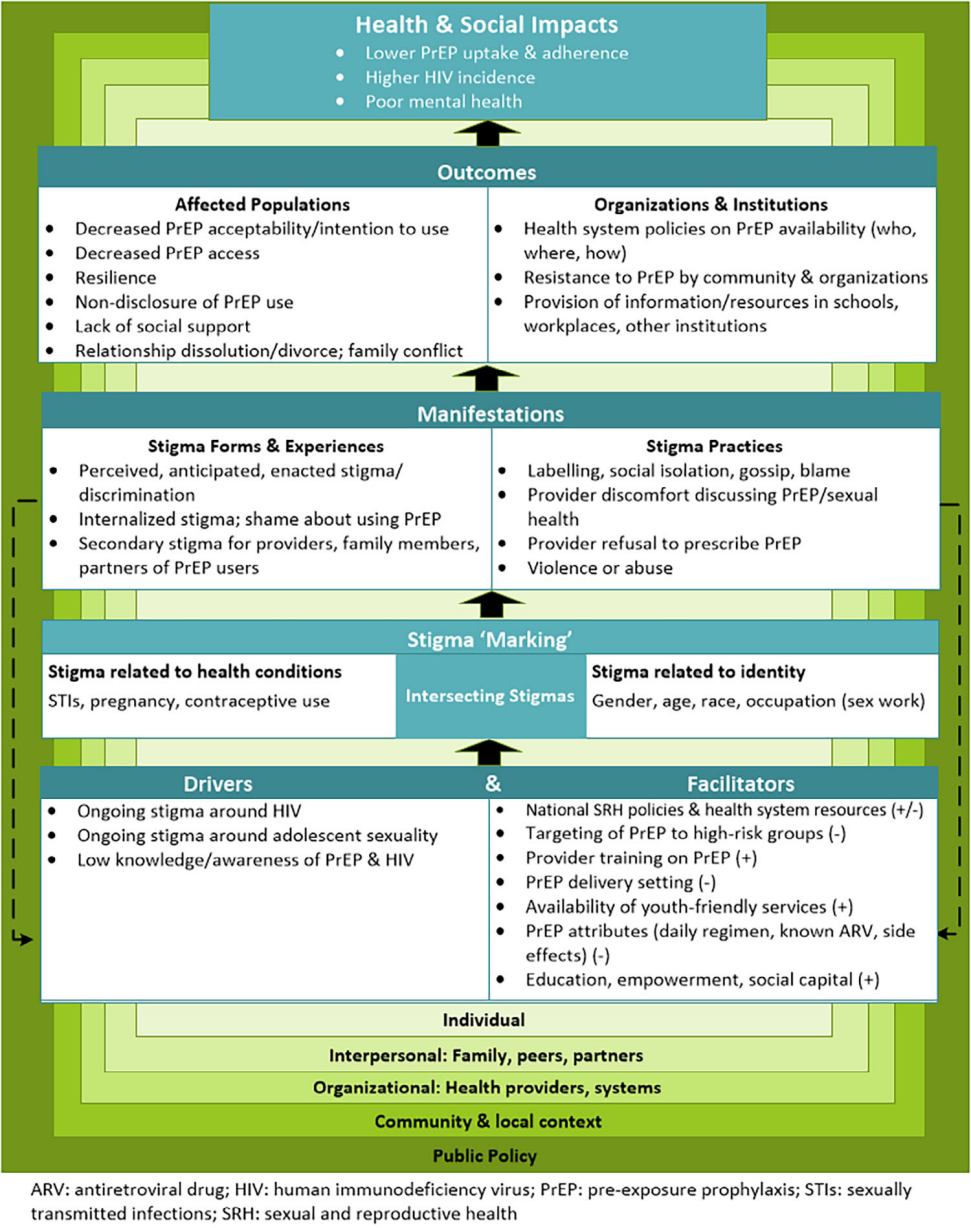
Consolidated PrEP stigma conceptual framework.

The final framework identifies (1) drivers; (2) facilitators; (3) manifestations of PrEP stigma related to AGYW; (4) intersecting stigmas; and (5) direct outcomes and health impacts. Throughout the results, we elucidate key findings from the three data sources that informed each domain. Figure 2 displays a reduced set of factors across sources. Table 1 includes all identified factors and their sources and serves as a source reference of each theme described below.

**Table 1:** Table of all identified PrEP stigma factors by conceptual framework level and source

	Source		
Factor	Literature review	Expert consultations^a^	FGDs
*Drivers*			
*HIV stigma*			
Fear of being seen as HIV positive	✓	✓	✓
PrEP mistaken for ARVs	✓	✓	✓
Being seen at HIV clinic to access PrEP	✓		
Belief that HIV is a punishment for immoral sexual behaviour	✓		
Fear of HIV testing, required for PrEP	✓		✓
*Sexuality stigma*			
PrEP is a sign of and associated with immoral or socially unacceptable sexual behaviours	✓	✓	✓
Labels or perceptions of “promiscuity” pertaining to premarital sex, sex work, multiple partnerships, *infidelity*, disreputable (risky) partners and sexual appetite	✓		✓+
Labelling adolescent sexuality as unacceptable in and of itself	✓		✓
PrEP is a cause of bad behaviour, that is risk compensation			✓
Associated with violation of traditional female gender norms of chastity, fidelity, obedience, docility	✓	✓	
PrEP less virtuous than condoms, associated with irresponsibility	✓		✓
*Lack of knowledge about PrEP among community members and potential users*			
Rumours around/fear of side effects and toxicities, especially infertility; belief that PrEP causes malaria and other diseases; PrEP is family planning; PrEP reduces sex drive	✓		✓
Lack of awareness that PrEP exists	✓		✓
Concerns about effectiveness, ARV resistance	✓		
Caveat: knowledge about PrEP targeting/eligibility requirements (when applicable) could increase stigma	✓		
Fear of ramifications of stereotypes and prejudices (see Stigma Practices)	✓		✓
*Facilitators (Positive and negative)*			
*Individual level*			
Marital status (+/−)	✓		X
Higher education levels among AGYW (+)	✓		✓
Empowerment/self‐efficacy (+)	✓	✓	✓
DREAMS participation (+)		✓	✓
Higher degree of privacy in the household (+)	✓		
Personal relationships with someone living with HIV, *including fear of losing a family member due to HIV* (+)	✓		✓+
Social support and acceptance of PrEP use (+)	✓		✓
Other coping resources (+)	✓		
*Provider and health systems*			
Availability of youth‐friendly services; healthcare satisfaction (+)	✓	✓	✓
Resource availability or constraints	✓		
Provider perception of degree of burden to provide PrEP (−)	✓	✓	
Factors minimizing ability to build patient‐provided rapport (e.g. visit length and provider consistency) (−)	✓		✓
Low degree of confidentiality in health facilities (−)	✓		✓
Cost and availability of PrEP (+/−)	✓	✓	
Degree of provider knowledge of and training in PrEP provision	✓	✓	✓
More understanding of toxicity, resistance and efficacy (+)	✓		
Qualifications required to receive training on provision of PrEP (−)	✓		
Improved level of support and advice from healthcare workers (+)			✓
Enhanced provider training in discussing sexual health or understanding stigma (+)	✓	✓	
PrEP delivery settings, for example ARV clinics, community and home settings, within support clubs (+/−)	✓	✓	✓
Targeting of PrEP and restriction of eligibility towards “high risk” groups (−)	✓	✓	
*Community*			
Religiosity, including objection to any medication (−)	✓		✓
Exposure to community outreach campaigns about PrEP and other effective PrEP messaging (+)	✓		✓
Higher exposure to other women using PrEP (e.g. PrEP champions/ambassadors) (+)	✓	✓	✓
Low education levels among community members (ability to understand about PrEP) (−)	✓		✓
Lack of knowledge about HIV transmission, care and treatment, and sexual health (−)	✓		
Deeply ingrained “medical mistrust” (−)	✓		✓
Inherent desire to protect youth's health (among adults) (+)	✓		
Belief/observation that PrEP works (e.g. in case of wife inheritance and serodiscordent couples where HIV‐negative woman takes PrEP and remains negative) (+)			✓
Strongly held gender norms around AGYW's sexuality and agency	✓		
Limit access to information and decision‐making power (−)	✓		
AGYW seen as irresponsible and immature, not ready for sex (−)	✓		
Belief that they drive sexuality (see “Drivers”) (−)	✓		
Importance of fertility and childbearing (−)	✓		
Norms around AGYW's lack of privacy (e.g. at community level) (−)	✓		
Social status/capital of youth in communities (+/−)	✓	✓	
*Legal/Policy*			
Older age of consent for HIV testing or PrEP and need for parental consent (−)	✓		
Lack of comprehensive sexual education in schools (−)	✓		
Lack of workplace policies on HIV testing (and potentially PrEP screening) (−)	✓		
Clarity and consistency of national policies for rollout (+)	✓	✓	
*PrEP attributes*			
Daily regimen (−)	✓		
Side effects (−)	✓		✓
Easily recognized as and branded as an ARV (−)	✓	✓	✓
*Intersecting stigma*			
Age	✓		✓
Gender/transgender identity	✓	✓	
Sex worker	✓	✓	✓
Race	✓	✓	
Socio‐economic status (*poverty, education level*)	✓		✓+
Drug/substance use, including alcohol	✓		✓
Sexual orientation	✓		
STIs	✓		
Teenage or unwanted pregnancy	✓	✓	✓
*Manifestations—stigma experiences*			
*Individual level*			
*Fear, anticipation or experience of stigma practices (as described below)*			
Need for secrecy; fear associated with disclosure	✓		✓
*Internalized*			
Self‐blame/shame/negative self‐image	✓	✓	✓
*Community and interpersonal levels*			
*Secondary (Experienced by these key influencers, or anticipated by the influencer or the participant)*			
Relationship partners feel threatened, mistrusted, disrespected	✓	✓	✓
Family members blamed and stigmatized for AGYW's need for PrEP or for allowing her to use PrEP	✓	✓	✓
Providers blamed and stigmatized for prescribing PrEP to AGYW	✓	X	✓
*Manifestations—stigma practices*			
*Negative attitudes and practices*			
*Community and interpersonal levels*			
Judging PrEP user as HIV positive, irresponsible, sexually amoral	✓	✓	✓
Social isolation and rejection (*parents warn children not to associate with PrEP users*)		✓	✓+
Name calling, *insulting* and labelling; *stereotyping*	✓	✓	✓+
Blaming (*for partner's HIV status*)	✓		✓+
Gossiping (*including disclosing to others without permission*)	✓	✓	✓+
Distrust by partners and family members	✓		✓
Actively discouraging or opposed to AGYW PrEP use (*under guise of religious reasons among church members*)			✓+
Violence and abuse (physical, sexual, verbal, economic)—*may also be outcomes*	✓	✓	✓
Physical punishment for sexual activity on PrEP use	✓		✓
Verbal harassment from healthcare workers	✓		✓
Neglect			✓
*Provider and health systems*			
Discomfort discussing PrEP	✓		✓
Discomfort providing PrEP for those not “high risk”	✓		
Fear of risk disinhibition with PrEP use	✓		✓
Discouraging PrEP use, expressions of disappointment, scolding	✓		✓
Rude, disrespectful or discriminatory behaviour towards AGYW seeking SRH services	✓		✓
Imposing conditions for service provision (e.g. requiring accompaniment of parent or spouse/sexual partner)	✓	✓	
Not keeping confidentiality	✓		✓
Denying access to services	✓		✓
Unwilling to prescribe PrEP to those not at classified as high risk according to set procedures/policies	✓		
Unwilling to prescribe for fear of risk compensation	✓		✓
*Outcomes—affected populations*			
*Individual level*			
Decreased PrEP acceptability/interest/intention to use	✓	✓	✓
Direct effect of stigma (i.e. desire to avoid it)	✓	✓	✓
Belief in myths and misperceptions (e.g. infertility)			✓
Reduced risk perception	✓		✓
Unwillingness to test for HIV (in some cases)	✓		✓
Non‐disclosure of PrEP use (hidden/covert use)	✓		✓
Anxiety and/or guilt around non‐disclosure	✓		✓
Decreased agency and motivation (to use PrEP and to discuss it with others)	✓		✓
Lack of social support for PrEP use	✓		✓
Self‐isolation to avoid/reduce stigma	✓		✓
School absences/drop‐out			✓
Relationship dissolution/divorce; family conflict			✓
Resilience	✓	✓	✓
AGYW empowered to take care of themselves	✓	✓	✓
Self‐efficacy to negotiate/convince family and others to support use *or take PrEP themselves*	✓	✓	✓+
Creating a sense of community around PrEP		✓	✓
Higher sexual risk behaviour (due to mental health consequences or perceived invulnerability)	✓	✓	✓
*Provider and health systems*			
Decreased PrEP access (from provider challenges, *unwilling to go to clinic, change clinics*) and disparities in access	✓		✓+
*Outcomes—organizations and institutions*			
Healthcare worker policies and trainings on PrEP	✓		✓
Who should be assessed for PrEP or offered PrEP, and how (e.g. risk assessment procedures)	✓		
Policies on initiating and re‐starting PrEP	✓		
Decreased confidence of providers and poor service delivery	✓	✓	✓
Alignment of PrEP with more complicated ARV provision	✓	✓	
Difficulties in mobilizing community leaders and organizations to support PrEP	✓	✓	
Institutional (workplace, schools) support for PrEP	✓		
Provision of information about PrEP to employees/students	✓		
Decreased PrEP availability (because of mandates around where and how many locations provide it)	✓	✓	
Lack of buy‐in from policy leaders (at national, subnational and facility level)	✓	✓	✓
On‐site provision of PrEP services	✓		
*Individual and population health and social impacts*			
Low PrEP uptake and adherence *and continuation*	✓	✓	✓+
HIV incidence	✓		
Quality of life (Social Inclusion/Relationship quality)	✓		✓
Poor mental health (*feeling helpless and afraid; suicidal intentions; psychological distress*)	✓	✓	✓+
Injury (resulting from violence)	✓	✓	✓
Mortality (e.g. suicide)			✓

*Note*: Where possible, the factors are organized by level of the socio‐ecologic framework. However, some factors, especially those related to other forms of stigma, inherently operate at multiple socio‐ecological levels and thus we did not attempt to assign them to a single level.

Abbreviations: AGYW, adolescent girls and young women; ARVs, antiretrovirals; PrEP, pre‐exposure prophylaxis; SRH, sexual and reproductive health.

^a^ Key informant interviews included review of the conceptual framework based on the literature review. Therefore, this column indicates items where experts raised explicit contributions such as emphasizing a factor, rather than checking all factors broadly agreed with at each level.

+ Indicates those items where FGD participants contributed additional details to an existing item, the additional details are italicized.

X Indicates items where FGD participants explicitly contradicted the contribution of an item, for example marital status was viewed as non‐protective.

### 3.1 Drivers

Three primary drivers of PrEP stigma were identified: ongoing and intersecting stigma around HIV (#1) and around adolescent sexuality (#2), as well as low PrEP awareness (#3).

#### 3.1.1 HIV stigma

While there were mixed opinions about whether HIV stigma has declined, fear of being seen as HIV positive and potential resulting stigma continue to be strong drivers of PrEP stigma [22, 41, 42, 43]. The literature described this as a carryover effect: because PrEP is an antiretroviral (ARV), it is often viewed as “HIV medication” even if the user is HIV negative [44]. This association extends to the public realm where if a user is seen attending an HIV‐focused clinic [22, 45], where PrEP is often dispensed, or is seen to carry or take pills that appear as ARVs [43], they will be thought to be HIV positive, for example “*if someone sees you with the bottle, she thinks that you are on ARV drugs because their color looks alike…and also they are taken daily’.”* [FGD2, PrEP users]. Fear of being labelled as HIV positive also negatively impacted HIV testing required to access PrEP:

*“They (AGYW) can go through stress when she thinks that the community knows that she is using ARV not PrEP and she has HIV and…she never wanted to go to the hospital to be tested.”* [FGD2, PrEP users]

#### 3.1.2 Sexuality stigma

Sexuality stigma was another significant driver of PrEP stigma, and intersected with HIV stigma, as PrEP use was associated with immoral sexual behaviours for AGYW like sex work, infidelity, and having multiple and/or risky sexual partners [15, 31, 43, 44, 46]. This was described frequently by PrEP users and influencers alike as permeating community‐held beliefs about PrEP:

*“When their boyfriends realize that their girlfriends are taking PrEP they will say that their girlfriends have multiple sexual partners and they are taking ARVs. Also, the parents of these girls realize that their daughters are taking PrEP, but the parents will say that the girls are prostitutes.”* [FGD2, PrEP users]

Particularly among HCPs, sexuality stigma also led to the belief that PrEP use could *cause* immoral sexual behaviour through risk disinhibition [14, 47, 48]. One review article concluded that PrEP was seen as less virtuous than condoms [43], suggesting that PrEP users are more irresponsible than those engaging in condom‐protected sex. YABs agreed that providers would rather talk about anything, including condoms, than PrEP because of their belief that PrEP encourages risk behaviours. As one provider said, they will *“judge you like you are going now on to trouble.”* [FGD1, HCPs]

Finally, while not often explicitly described as separate from sexuality stigma, one expert exclaimed, “*gender underpins everything”* pointing towards the role of gender norms in beliefs around acceptable sexual behaviour for AGYW. This was discussed in more depth with one YAB, whose members described the unsuccessful attempt of a Kenyan organization to market PrEP as something that gives AGYW “power.” The youth explained the message did not resonate with them because they felt PrEP could not overcome AGYW's sense of powerlessness in a society where their partners and parents make all decisions.

#### 3.1.3 Low PrEP knowledge

The third major contributor to PrEP stigma was low awareness and understanding of PrEP in communities which contributed to misidentifying PrEP as being for people living with HIV. In addition, misconceptions around PrEP effectiveness [49] and side effects were identified as contributing to stigma in the literature and FGDs. As one PrEP user explained, community members discourage AGYW from using PrEP because they believe it can cause “*complications during birth….it can interfere with your womb”* [FGD4, PrEP users]. Adding to this, YAB members described how providers fear PrEP would cause side effects, but also violence, and, therefore, prefer to recommend other forms of prevention like abstinence or condoms.

### 3.2 Facilitators

While facilitators can be either negative or positive influences on PrEP stigma, this section presents “positive” facilitators, or factors that combat PrEP stigma when positively valanced. Most facilitators were identified in the literature, and experts and FGD participants supported findings at the health system, community and individual levels of the socio‐ecological framework, but did not touch on policy facilitators. Key facilitators raised across sources include the positioning and provision of PrEP, engagement with existing social and communication networks, and individual empowerment.

#### 3.2.1 Positioning of PrEP

Crossing the policy and healthcare levels, positioning PrEP as a service that is separate from HIV services and not only for high “risk” individuals were critical facilitators to combatting PrEP stigma. Several experts noted that careful consideration of where and how PrEP is provided could reduce stigma. For example, separating PrEP from HIV services and integrating it into youth‐friendly services, as well as positioning PrEP counselling as a discussion of an individual's needs and goals around HIV prevention, rather than identification of “risk” factors, could shift broader negative attitudes. This shift, if embedded into provider training, could further improve providers’ engagement with youth, focusing on positive aspects of PrEP rather than lecturing about sexuality.

#### 3.2.2 Social support

A second category of facilitators, at the community and interpersonal levels, was the enhancement of existing social support through community outreach and engagement of youth as ambassadors (i.e. sources of support and knowledge). Literature and experts highlighted the strong social capital of AGYW [50], which could be channelled into programmes like PrEP ambassadors. Similarly, channelling knowledge and social support in broader community networks may combat PrEP stigma, as was achieved for HIV [51, 52]. Exposure to PrEP users and PrEP success stories, similar to “contact” programmes used to combat HIV stigma [53], was viewed as facilitating positive perceptions. FGD participants highlighted instances where community members saw HIV‐negative members of sero‐different couples remain HIV negative and, therefore, began to believe in PrEP effectiveness.

*“Let's pretend I don't take medicine and my husband takes medicine (i.e. ARVs). And have come and told others the importance of PrEP…they see I am not…HIV positive and my partner is positive. I think…they can know the importance of PrEP.”* [FGD2, Family members]

#### 3.2.3 Empowerment

In contrast to the marketing campaign described above that sought to message PrEP as empowering, experts and FGD participants identified substantive efforts to empower AGYW as a way to reduce PrEP stigma. Experts connected this to the creation of high‐status roles associated with PrEP, such as the aforementioned “youth ambassador” concept. YAB members felt that girls were born with the confidence and skills to enact autonomous decisions. However, they acknowledged that these attributes may need to be bolstered through interventions like assertiveness trainings, or employment connected to ambassador roles. FGD participants described personal experiences where the PrEP knowledge they gained through DREAMS helped them respond to others’ concerns or access support to be resilient in PrEP use despite others’ scepticism.

*R5: Whenever my friends and family heard the sound or noise from the bottle containing PrEP they would ask me what it was. I told them they are PrEP, the drugs that prevent you from getting HIV. They said that this HIV can only be treated, not prevented and [asked] where I got the drugs from. I told them that it is [the DREAMS implementor] who gave us and told us that they are drugs preventing HIV virus and they are not ARVs*.

*I: I see and how did this affect you?*

*R5: I was not affected because I knew what I wanted for my health…I just went on with PrEP*. [FGD2, PrEP users]

The literature identified numerous additional individual characteristics as being protective against stigmatizing attitudes and reducing perceived stigma, including marriage, higher education levels [54], older age and high perceived social capital [50]. While FGD participants reported that education and knowledge may empower young women in their PrEP use, they thought being married might increase stigma by raising more questions about why one was using PrEP.

### 3.3 Intersecting stigmas

Based on our literature review, PrEP stigma may intersect with stigma related to sexual health, such as pregnancy, contraceptive use and sexually transmitted infections (STIs) and stigma related to other marginalized identities based on gender (both female and transgender), age, race, sexual orientation and occupation (especially sex work) [20, 31, 42, 43, 44, 55, 56]. Identity‐based stigma is known to intersect with HIV stigma [50, 52, 57] because of elevated HIV prevalence among MSM, transgender women and people who sell sex. For AGYW, especially those who are unmarried, the stigma around sexual and reproductive health behaviours and conditions and PrEP stigma share a common driver in the taboo around women's sexuality [58, 59, 60, 61, 62]. This is exacerbated by the belief that PrEP use promotes immoral and risky sexual behaviour, leading to higher rates of unintended pregnancy, contraceptive use and STIs. Although HIV and sexuality stigma could also be categorized as intersecting stigmas, we followed experts’ recommendations to categorize them as drivers due to their primary role in creating PrEP stigma.

Experts also highlighted the importance of socio‐economic status (SES), including poverty and education levels, as intersecting stigmas. The desire to avert both poverty and its resulting stigma may encourage young women to engage in transactional sex, exposing them to stigma from sex work or being labelled “promiscuous.” Conversely, the community may rationalize sex work and transactional sex among poor women as necessary for survival, but may stigmatize those of higher SES for being sexually active when they do not “need” to be. In consultations and FGDs, Kenyan AGYW and key influencers also highlighted intersecting stigmas due to pregnancy and sex work. One PrEP user said *“*[elderly women*] take it that* [PrEP] *prevents HIV but it opens the way to prostitution, which is the reason why she will warn her granddaughter not to get closer to me because I might introduce her into prostitution.”* [FGD1, PrEP users]

Participants also introduced a new intersection with stigma around alcohol use, which was discussed in multiple FGDs, but had not been identified through the literature review or expert consultations. This intersection arose from assumptions that it was unhealthy to use PrEP with alcohol, and from the underlying associations of PrEP and alcohol use with sexuality. One healthcare worker described, *“So if you are taking alcohol at the same time you are taking PrEP, they will know you are involved in transactional sex.”* [FGD3, HCPs]

### 3.4 Manifestations

The domain of manifestations is broken into stigma practices–beliefs, attitudes and actions that generate and reinforce stigma–and experiences of stigma, which are the lived realities of stigmatized people. For PrEP, all sources found that PrEP stigma manifests in similar ways to HIV and sexuality stigma, through labelling, shaming and occasionally violence.

#### 3.4.1 Practices

Informed by the literature, PrEP stigma practices were divided into those occurring at the community/interpersonal and healthcare levels, with many common practices across the two.

At the healthcare level, literature cited provider reluctance to prescribe PrEP or restricting PrEP to specific categories of people such as sero‐different couples [14, 43, 55]. PrEP users were labelled as irresponsible and sexually amoral, and relatedly shamed [54, 57, 58, 60, 63] and subject to verbal abuse [44].

At the community and interpersonal level, literature noted that PrEP users are blamed and called names [14, 49], distrusted by partners [44] and judged as being HIV positive [42]. Drawing from HIV and sexuality stigma, additional practices could include social isolation/rejection [54, 56, 57, 60], and exposure to verbal violence [64]. FGDs with PrEP users and key influencers agreed with these findings, providing greater nuance to practices such as social isolation and rejection, such as parents warning their children not to associate with PrEP users.

*“They* [parents] *will warn you not to associate with her that she will engage you in immoral behaviors*.” [FGD4, AGYW peers]

Experts and FGD participants both elaborated on the role of violence. Experts felt that intimate partner violence should be highlighted more clearly among stigma practices. Participants in all FGDs described violence, in various forms, as a manifestation. This was often linked to non‐disclosure or inadvertent disclosure, that is someone else disclosing their PrEP use without permission.

*“…the information went around, and the mother‐in‐law got the information. The mother‐in‐law might cause violence to the young couple and can ruin their marriage.”* [FGD1, Family members]

#### 3.4.2 Experiences

Literature suggested that experiences of stigma include not only direct experiences of gossip or isolation, but also fear or anticipation of the previously mentioned practices [24, 65]. Internalized stigma (e.g. feeling self‐blame, shame and having a negative self‐image) [47, 54], and secondary stigma experienced by partners, family members or providers through association [56, 66] were also identified as important stigma forms.

Experts and FGD participants generally supported the literature, although experts felt that evidence of secondary stigma among HCPs has yet been demonstrated, while FGDs with family members confirmed concerns about secondary stigma. For example, family members suggested that parents worry about gossip and being blamed for “spoiling” their children or encouraging prostitution.

*“So they will say this person is doing a business with her daughter because she has allowed her daughter to use PrEP and those who take it are prostitutes. That means she has allowed her daughter to engage with multiple partners and then bring her some money.”* [FGD2, Family members]

Experts also emphasized the greater impact of internalized stigma over perceived or experienced stigma on PrEP use; however, in FGDs, internalized stigma only came up occasionally among PrEP users themselves, and was described as a sense of shame.

### 3.5 Outcomes

#### 3.5.1 For affected populations

In the limited literature focused on PrEP, outcomes of stigma included lower PrEP acceptability, less interest and intention to use it, discomfort discussing PrEP with providers and less willingness to prescribe PrEP by providers [23, 31, 46, 47]. Hall et al. reported similar outcomes for contraceptive use because of the sexuality stigma around AGYW in Ghana [54]. In Kenya [53], higher levels of HIV stigma were associated with reduced willingness to test for HIV, which would decrease PrEP use.

Other outcomes documented in HIV and sexuality stigma literature included lack of disclosure [65], lack of social support [67], and disruption of education, employment, relationship and living situations [54]. Experts and FGD participants reinforced the salience of these themes. Experts added that AGYW may face pressure from sexual partners to engage in condomless sex, increasing sexual risk. All FGD participant categories discussed how consequences of PrEP stigma included relationship dissolution and divorce, and one PrEP user described how it could lead to school absences or drop out.

*“But in their school, they do not allow you to take PrEP…So they are going through challenges, so if the teachers have really made her life difficult like that, so she will also just decide to drop out of school.”* [FGD4, PrEP users]

Finally, psychological stress and discomfort [67], as well as resilience [18, 44, 65] came up in the HIV literature and among FGD participants. An HCP described how being perceived as HIV positive creates discomfort while seeking PrEP.

*“She is being ushered towards the pharmacy and there she will find other clients who are also queuing for their ARVs so she becomes uncomfortable, if you don't follow‐up she might just leave without the pills.”* [FGD2, HCPs]

However, the discomfort could be overcome by the desire to live HIV‐free [18, 44, 65], which FGD participants said could be enhanced by encouragement from other PrEP users or providers.

*“They just continue encouraging each other…With the neighbors that are also taking PrEP…Let us just have the courage and continue using PrEP…Even if people are talking about…Because they know PrEP…has prevented them from HIV.”* [FGD3, PrEP users]

#### 3.5.2 For organizations and institutions

Outcomes at the organizational level are less documented and did not come up in FGDs. Literature from the HIV field suggests that stigma can promote discriminatory policies and laws, such as the criminalization of transmission [37, 52], and make it difficult to mobilize community‐wide intervention [60]. Experts elucidated how policies such as those aligning PrEP provision with HIV service provision reduce confidence among providers and create fear among them that PrEP provision requires high levels of training and could otherwise lead to harmful outcomes. They also described how these policies limit the accessibility of PrEP due to limits on who, how and where it can be provided.

### 3.6 Impact

The ultimate impact of stigma is non‐uptake, non‐adherence or discontinuation of PrEP use. Several reviews and qualitative studies suggested that stigma may impact these behaviours [12, 18, 21, 24, 43, 44, 49], and one quantitatively documented an impact [68]. Experts and FGD participants agreed with these conclusions. Other serious impacts included depression, suicide and violence‐related injury. These were described in the literature [52, 62, 66, 67, 69] and by experts and FGD participants. Not surprisingly, mental health and PrEP discontinuation were intimately connected, with depression leading to discontinuation.

“*Emotionally they are down…They are psychologically tortured…majority discontinue PrEP use to prove that they are not taking ARVs.”* [FGD3, PrEP users]

## 4 DISCUSSION

Using the established HSDF [37] and a multi‐stage, multi‐stakeholder process, we developed a comprehensive PrEP stigma conceptual framework, which can be used to understand and further investigate this topic. The framework outlines how and why PrEP stigma exists among AGYW in sub‐Saharan Africa, its intersectional nature, how it manifests, and the variety of outcomes and impacts for both individuals and within the health system. Over the period during which this framework was developed, additional research has emerged. To ensure the current relevance of our model, we re‐ran our literature search in March 2023, finding seven articles which explored PrEP stigma among AGYW in African contexts [25, 26, 27, 70, 71, 72, 73, 74] and seven that developed PrEP stigma scales [28, 29, 75, 76, 77, 78]. Findings from these studies aligned with our model factors, confirming its contemporary relevance. They also highlighted an ongoing gap, in that only two studies focused research on the health sector [70, 74], and none used a conceptual framework.

The study's method of triangulation and participatory end‐user processes provide strength to the findings. Designed to elucidate issues of PrEP stigma experienced by AGYW in sub‐Saharan Africa, the initial framework drew from global literature on HIV, sexuality and PrEP stigma across different country contexts, and follow‐on phases confirmed and refined the model. The strength of this model suggests its relevance as a framework for ongoing research with AGYW and other populations. In addition, participatory end‐user‐engaged research is critical to the quality of research outputs and benefits to the community [79]. Not only did our work include consultations with youth experts in the second phase, but we also involved a YRT in the conduct and interpretation of FGDs in the third phase. These processes raised important distinctions specifically for Kenyan AGYW, such as marriage as a negative facilitator to PrEP stigma, the intersectional issue of alcohol use, and the nature and potential severity of additional outcomes, for example relationship dissolution. Relationship dissolution has since been identified in relation to PrEP stigma in another study in Tanzania [25].

By articulating the domains of the stigmatization process and providing a comprehensive description of each domain, the framework can help researchers identify potential intervention targets and mechanisms of action. Interventions seeking to ***reduce PrEP stigma*** will need to combat HIV and sexuality stigma, and increase community‐wide knowledge of PrEP in a non‐stigmatizing way, for example by framing it as a tool for anyone to reduce HIV anxiety or protect one's sexual health rather than for people at high risk of HIV acquisition [12]. The empowerment of PrEP users and their integration into educational campaigns may strengthen the effectiveness of these efforts. AGYW in recent PrEP trials describe how counselling and peer support interventions improved their PrEP knowledge and disclosure skills and empowered them to combat stigma and rumours [72, 80]. Additionally, core shifts at the healthcare level, such as integrating PrEP provision with general youth health rather than HIV treatment, and changing language around who should use PrEP away from “risk” and towards sexual wellbeing, will facilitate stigma reduction. Interventions aiming to ***mitigate PrEP stigma outcomes*** *and impacts* among AGYW using PrEP should aim to build resilience and social support from peers, partners, family and/or counsellors, and provide counselling to address strategies to support undisclosed PrEP use or address consequences of involuntary disclosure. These approaches are not novel [12, 25, 46, 81, 82, 83, 84, 85, 86]; however, the framework helps identify the expected impacts of each approach on stigma. This, in turn, can help ensure that evaluation efforts measure change in the domains their intervention targets, rather than domains further up—or downstream in the process, a common limitation in previous studies [87]. The framework can also help researchers develop or select measures of PrEP stigma among AGYW that include factors most relevant to this population [88].

Despite the rigour of our process, this framework is limited by our selection of organizations, institutions and settings participating in its development. Experts were primarily based in the United States, Kenya and South Africa, and participation may have been influenced by the study team's network; FGDs were limited to youth in Siaya County, Kenya. We also focused exclusively on oral PrEP, because it was the only formulation available, but the role of stigma may differ for other formulations in the future. We did, however, seek to identify global experts in these fields, and include youth from urban, peri‐urban and rural Kenya, and the triangulation of our results across multiple sources, including literature from a wide range of countries, offers support that the findings may be relevant beyond AGYW in Kenya. Few FGD participants offered insights on the organizational and institutional level outcomes suggested by the literature, limiting our ability to understand their applicability to the local context.

Finally, decades of work in HIV stigma and discrimination suggests applying an intersectional lens [89, 90] to PrEP stigma research, and interventions will be critical [91, 92, 93]. Berger first coined the term “intersectional stigma” [94] to describe the process through which Black American women living with HIV face HIV stigma that intersects with discrimination related to race, class and gender, but also with behaviours, such as drug use or sex work. As noted previously, we categorized intersecting stigmas of HIV and AGYW sexuality as drivers given their critical role in PrEP stigma as suggested by experts and confirmed by all sources. Yet, other intersecting stigmas based on factors such as gender‐identity, sexuality, poverty and other sexual health issues such as pregnancy and STIs, which were mentioned in the literature and expert interviews, were largely not confirmed in discussions with youth. The omission of gender‐identity and sexuality, in particular, may reflect the marginalized status of gender and sexual minority youth in Kenya, and the fact that we did not intentionally sample these populations because our research focused on cisgender heterosexual AGYW. However, the role of these intersecting stigmas, including poverty, and alcohol use, should be further explored in studies with diverse populations. Research tools that more specifically investigate issues of intersectional stigma, as have been used in research with young Black American women's PrEP use [95], should also be considered.

## 5 CONCLUSIONS

Our PrEP stigma conceptual framework highlights potential intervention targets at multiple levels of influence and points in the stigmatization process. Adoption of this framework would enable researchers to develop standardized measures and compare the burden of stigma across timepoints and populations as well as evaluate intervention outcomes.

## COMPETING INTERESTS

The authors declare that they have no competing interests.

## AUTHORS’ CONTRIBUTIONS

STR, MH, LN and KA conceived of the research study, obtained funding and designed the research study. SO, TM and KA implemented the research. STR and MH conceptualized this research question. MH, STR, TM and SO conducted this analysis, and MH and SR drafted the manuscript. All authors have reviewed and approved the final manuscript.

## FUNDING

This research was supported by the Fogarty International Center (FIC), the Eunice Kennedy Shriver National Institute of Child Health & Human Development (NICHD) and the National Institute of Mental Health (NIMH) of the National Institutes of Health (R21TW011784). The study was also funded by RTI International through an Independent Research & Development award.

## DISCLAIMER

The content is solely the responsibility of the authors and does not necessarily represent the official views of the National Institutes of Health or RTI International.

## Supporting information

File S1: Literature review search strategy
Information on file format. Document outlining the literature search terms used for all databases.

## Data Availability

Data are available on request from the authors.

## References

[1] UNAIDS . Global AIDS update 2019. Communities at the centre: defending rights, breaking barriers, reaching people with HIV services. Geneva, Switzerland: UNAIDS; 2019.

[2] Koss CA , Charlebois ED , Ayieko J , Kwarisiima D , Kabami J , Balzer LB , et al. Uptake, engagement, and adherence to pre‐exposure prophylaxis offered after population HIV testing in rural Kenya and Uganda: 72‐week interim analysis of observational data from the SEARCH study. Lancet HIV. 2020;7(4):e249–e261. 32087152 10.1016/S2352-3018(19)30433-3 PMC7208546

[3] Rousseau‐Jemwa E , Bekker L‐G , Bukusi E , Delaney‐Moretlwe S , Omollo V , Travill D , et al. Early persistence of HIV pre‐exposure prophylaxis (PrEP) in African adolescent girls and young women (AGYW) from Kenya and South Africa. HIV Research for Prevention Conference; 21–25 October, 2018; Madrid, Spain.

[4] Kyongo JK , Kiragu M , Karuga R , Ochieng C , Ngunjiri A , Wachihi C , et al. How long will they take it? Oral pre‐exposure prophylaxis (PrEP) retention for female sex workers, men who have sex with men and young women in a demonstration project in Kenya. J Int AIDS Soc. 2018;21(Suppl 6):e25148.30051631

[5] Tapsoba JDD , Zangeneh SZ , Appelmans E , Pasalar S , Mori K , Peng L , et al. Persistence of oral pre‐exposure prophylaxis (PrEP) among adolescent girls and young women initiating PrEP for HIV prevention in Kenya. AIDS Care. 2020;33(6):712‐720 32951437 10.1080/09540121.2020.1822505 PMC7981281

[6] Celum C , Gill K , Morton J , Stein G , Myers L , Thomas K , et al. Incentives conditioned on tenofovir levels to increase adherence among young women on PrEP in Cape Town. 10th IAS Conference on HIV Science; July 21–24, 2019; Mexico City, Mexico.

[7] Celum C , Hosek S , Tsholwana M , Kassim S , Mukaka S , Dye BJ , et al. PrEP uptake, persistence, adherence, and effect of retrospective drug level feedback on PrEP adherence among young women in southern Africa: results from HPTN 082, a randomized controlled trial. PLoS Med. 2021;18(6):e1003670. 34143779 10.1371/journal.pmed.1003670 PMC8253429

[8] Celum CL , Bukusi EA , Bekker LG , Delany‐Moretlwe S , Kidoguchi L , Omollo V , et al. PrEP use and HIV seroconversion rates in adolescent girls and young women from Kenya and South Africa: the POWER demonstration project. J Int AIDS Soc. 2022;25(7):e25962. 35822945 10.1002/jia2.25962 PMC9278271

[9] Sweeney SM , Vanable PA . The association of HIV‐related stigma to HIV medication adherence: a systematic review and synthesis of the literature. AIDS Behav. 2016;20(1):29–50. 26303196 10.1007/s10461-015-1164-1

[10] Turan JM , Bukusi EA , Onono M , Holzemer WL , Miller S , Cohen CR . HIV/AIDS stigma and refusal of HIV testing among pregnant women in rural Kenya: results from the MAMAS study. AIDS Behav. 2011;15(6):1111–1120. 20827573 10.1007/s10461-010-9798-5 PMC3127002

[11] Karim QA , Meyer‐Weitz A , Mboyi L , Carrara H , Mahlase G , Frohlich JA , et al. The influence of AIDS stigma and discrimination and social cohesion on HIV testing and willingness to disclose HIV in rural KwaZulu‐Natal, South Africa. Glob Public Health. 2008;3(4):351–365.

[12] Golub SA . PrEP stigma: implicit and explicit drivers of disparity. Curr HIV/AIDS Rep. 2018;15(2):190–197. 29460223 10.1007/s11904-018-0385-0 PMC5884731

[13] Golub SA , Lelutiu‐Weinberger C , Surace A . Experimental investigation of implicit HIV and preexposure prophylaxis stigma: evidence for ancillary benefits of preexposure prophylaxis use. J Acquir Immune Defic Syndr. 2018;77(3):264–271. 29140872 10.1097/QAI.0000000000001592

[14] Herron PD . Ethical implications of social stigma associated with the promotion and use of pre‐exposure prophylaxis for HIV prevention. LGBT Health. 2016;3(2):103–108. 26859191 10.1089/lgbt.2014.0114

[15] Braksmajer A , Senn TE , McMahon J . The potential of pre‐exposure prophylaxis for women in violent relationships. AIDS Patient Care STDs. 2016;30:274–281. 27286296 10.1089/apc.2016.0098 PMC4913495

[16] Mack N , Odhiambo J , Wong CM , Agot K . Barriers and facilitators to pre‐exposure prophylaxis (PrEP) eligibility screening and ongoing HIV testing among target populations in Bondo and Rarieda, Kenya: results of a consultation with community stakeholders. BMC Health Serv Res. 2014;14:231. 24886646 10.1186/1472-6963-14-231 PMC4051373

[17] Sawyer SM , Afifi RA , Bearinger LH , Blakemore SJ , Dick B , Ezeh AC , et al. Adolescence: a foundation for future health. Lancet. 2012;379(9826):1630–1640. 22538178 10.1016/S0140-6736(12)60072-5

[18] Velloza J . The influence of HIV‐related stigma on PrEP disclosure and adherence over time among AGYW in HPTN 082. IAPAC Adherence Conference; 17–19 June, 2019; Miami, FL.

[19] Wagner LD , Roberts ST , O'Rourke S , Celum C , Baeten JM , Bukusi E , et al. Challenges with oral pre‐exposure prophylaxis (PrEP) disclosure among adolescent girls and young women (AGYW) in Kenya and South Africa. AIDS Res Hum Retrov. 2018;34:373.

[20] Were D , Atkins K , Musau A , Plotkin M , Curran K . Manifestations of stigma in the context of a national oral pre‐exposure prophylaxis (PrEP) scale‐up programme in Kenya. J Int AIDS Soc. 2019;22:15.

[21] Amico KR , Wallace M , Bekker LG , Roux S , Atujuna M , Sebastian E , et al. Experiences with HPTN 067/ADAPT study‐provided open‐label PrEP among women in Cape Town: facilitators and barriers within a mutuality framework. AIDS Behav. 2017;21(5):1361–1375. 27317411 10.1007/s10461-016-1458-y PMC5378745

[22] Pintye J , Beima‐Sofie KM , Makabong OP , Njoroge A , Trinidad SB , Heffron RA , et al. HIV‐uninfected Kenyan adolescent and young women share perspectives on using preexposure prophylaxis during pregnancy. AIDS Patient Care STDs. 2018;32(12):538–544. 30036072 10.1089/apc.2018.0058 PMC6300042

[23] Vazquez L , Moll AP , Kacin A , Ndlovu NE , Shenoi SV . Perceptions of HIV preexposure prophylaxis among young pregnant women from rural KwaZulu‐Natal, South Africa. AIDS Patient Care STDs. 2019;33(5):214–219. 31067125 10.1089/apc.2018.0242 PMC6531897

[24] Stangl AL , Bryan C , Barre I , Lees S , Ramskin L , Khoza N , et al. The influence of stigma on PrEP uptake among adolescent girls and young women in Johannesburg, South Africa and Mwanza, Tanzania: qualitative findings from the EMPOWER study. AIDS Impact Conference; 29–31 July 2019; London. 10.1371/journal.pone.0339380 PMC12867226 41632776

[25] Jani N , Mathur S , Kahabuka C , Makyao N , Pilgrim N . Relationship dynamics and anticipated stigma: key considerations for PrEP use among Tanzanian adolescent girls and young women and male partners. PLoS One. 2021;16(2):e0246717. 33596216 10.1371/journal.pone.0246717 PMC7888654

[26] Bergam S , Harrison AD , Benghu N , Khumalo S , Tesfay N , Exner T , et al. Women's perceptions of HIV‐ and sexuality‐related stigma in relation to PrEP: qualitative findings from the Masibambane study, Durban, South Africa. AIDS Behav. 2022;26(9):2881–2890. 35218452 10.1007/s10461-022-03632-6 PMC9378426

[27] Munthali RJ , Stangl AL , Baron D , Barré I , Harvey S , Ramskin L , et al. Prevalence and risk factors of PrEP use stigma among adolescent girls and young women in Johannesburg, South Africa and Mwanza, Tanzania participating in the EMPOWER trial. AIDS Behav. 2022;26(12):3950–3962. 35776254 10.1007/s10461-022-03721-6 PMC9640431

[28] Atkins K , Kan L , Musau A , Reed J , Were D , Mohan D . Adaptation and psychometric evaluation of a scale to measure oral pre‐exposure prophylaxis‐related stigma among key and vulnerable populations in Kenya. J Int AIDS Soc. 2022;25(Suppl 1):e25929. 35818870 10.1002/jia2.25929 PMC9274213

[29] Budhwani H , Y İ , Maragh‐Bass AC , Rainer CB , Claude K , Muessig KE , et al. Development and validation of the youth pre‐exposure prophylaxis (PrEP) stigma scale. AIDS Behav. 2023;27(3):929–938. 36029425 10.1007/s10461-022-03829-9 PMC9968821

[30] Mustanski B , Newcomb ME , Ryan DT . PrEP stigma preducts PrEP uptake and adherence: results from the RADAR cohort study. Conference on Retroviruses and Opportunistic Infections (CROI); 4–7 March, 2019; Boston, MA.

[31] Calabrese SK , Dovidio JF , Tekeste M , Taggart T , Galvao RW , Safon CB , et al. HIV pre‐exposure prophylaxis stigma as a multidimensional barrier to uptake among women who attend planned parenthood. J Acquir Immune Defic Syndr. 2018;79(1):46–53. 29847480 10.1097/QAI.0000000000001762 PMC6092222

[32] Bil JP , Davidovich U , van der Veldt WM , Prins M , de Vries HJ , Sonder GJ , et al. What do Dutch MSM think of preexposure prophylaxis to prevent HIV‐infection? A cross‐sectional study. AIDS. 2015;29(8):955–964. 25915169 10.1097/QAD.0000000000000639

[33] Jackson T , Huang A , Chen H , Gao X , Zhong X , Zhang Y . Cognitive, psychosocial, and sociodemographic predictors of willingness to use HIV pre‐exposure prophylaxis among Chinese men who have sex with men. AIDS Behav. 2012;16(7):1853–1861. 22538373 10.1007/s10461-012-0188-z

[34] Golub SA , Fikslin RA , Goldberg MH , Pena SM , Radix A . Predictors of PrEP uptake among patients with equivalent access. AIDS Behav. 2019;23(7):1917–1924. 30600456 10.1007/s10461-018-2376-y PMC6571035

[35] Mustanski B , Ryan DT , Hayford C , Phillips G, 2nd , Newcomb ME , Smith JD . Geographic and individual associations with PrEP stigma: results from the RADAR cohort of diverse young men who have sex with men and transgender women. AIDS Behav. 2018;22(9):3044–3056. 29789985 10.1007/s10461-018-2159-5 PMC6076868

[36] Walsh JL . Applying the information‐motivation‐behavioral skills model to understand PrEP intentions and use among men who have sex with men. AIDS Behav. 2019;23(7):1904–1916. 30554396 10.1007/s10461-018-2371-3 PMC6571043

[37] Stangl AL , Earnshaw VA , Logie CH , Brakel WV , Simbayi LC , Barré I , et al. The Health Stigma and Discrimination Framework: a global, crosscutting framework to inform research, intervention development, and policy on health‐related stigmas. BMC Med. 2019;17(1):31. 30764826 10.1186/s12916-019-1271-3 PMC6376797

[38] Ferguson L , Gruskin S , Bolshakova M , Yagyu S , Fu N , Cabrera N , et al. Frameworks and measures for HIV‐related internalized stigma, stigma and discrimination in healthcare and in laws and policies: a systematic review. J Int AIDS Soc. 2022;25:e25915. 35818866 10.1002/jia2.25915 PMC9274352

[39] PEPFAR . DREAMS Overview: Kenya. 2015 [cited 8 December 2016]. Available from: http://www.pepfar.gov/documents/organization/253961.pdf.

[40] Roberts ST , Bann CM , Hartmann M , Otticha S , Nyblade L , Marton T , et al. Developing a validated scale to measure PrEP stigma among adolescent girls and young women in western Kenya. The 24th International AIDS Conference; 2022 29 July–2 August; Montreal, Canada.

[41] Calabrese SK . Interpreting gaps along the preexposure prophylaxis cascade and addressing vulnerabilities to stigma. Am J Public Health. 2018;108(10):1284–1286. 30207777 10.2105/AJPH.2018.304656 PMC6137772

[42] Rael CT , Martinez M , Giguere R , Bockting W , MacCrate C , Mellman W , et al. Barriers and facilitators to oral PrEP use among transgender women in New York City. AIDS Behav. 2018;22(11):3627–3636. 29589137 10.1007/s10461-018-2102-9 PMC6160363

[43] Haire BG . Preexposure prophylaxis‐related stigma: strategies to improve uptake and adherence—a narrative review. HIV AIDS (Auckl). 2015;7:241–249. 26508889 10.2147/HIV.S72419 PMC4610795

[44] Goparaju L , Praschan NC , Warren‐Jeanpiere L , Experton LS , Young MA , Kassaye S . Stigma, partners, providers and costs: potential barriers to PrEP uptake among US women. J AIDS Clin Res. 2017;8(9):730. 29201531 10.4172/2155-6113.1000730 PMC5708581

[45] Pinto RM , Berringer KR , Melendez R , Mmeje O . Improving PrEP implementation through multilevel interventions: a synthesis of the literature. AIDS Behav. 2018;22(11):3681–3691. 29872999 10.1007/s10461-018-2184-4 PMC6208917

[46] Pilgrim N , Jani N , Mathur S , Kahabuka C , Saria V , Makyao N , et al. Provider perspectives on PrEP for adolescent girls and young women in Tanzania: the role of provider biases and quality of care. PLoS One. 2018;13(4):e0196280. 29702659 10.1371/journal.pone.0196280 PMC5922529

[47] Calabrese SK , Underhill K . How stigma surrounding the use of HIV preexposure prophylaxis undermines prevention and pleasure: a call to destigmatize “truvada whores”. Am J Public Health. 2015;105(10):1960–1964. 26270298 10.2105/AJPH.2015.302816 PMC4566537

[48] Schwartz J , Grimm J . PrEP on Twitter: information, barriers, and stigma. Health Commun. 2017;32(4):509–516. 27295507 10.1080/10410236.2016.1140271

[49] Card KG , Hawkins BW , Mortazavi L , Gregory A , Ng KH , Lachowsky NJ . Stigma, the media, and pre‐exposure prophylaxis for HIV prevention: observations for enhancing knowledge translation and resisting stigma in the Canadian context. AIDS Behav. 2019;23(7):1877–1887. 30390190 10.1007/s10461-018-2332-x

[50] Cuca YP , Asher A , Okonsky J , Kaihura A , Dawson‐Rose C , Webel A . HIV stigma and social capital in women living with HIV. J Assoc Nurses AIDS Care. 2017;28(1):45–54. 27697368 10.1016/j.jana.2016.09.001 PMC5183462

[51] Asamoah CK , Asamoah BO , Agardh A . A generation at risk: a cross‐sectional study on HIV/AIDS knowledge, exposure to mass media, and stigmatizing behaviors among young women aged 15–24 years in Ghana. Glob Health Action. 2017;10(1):1331538. 28621223 10.1080/16549716.2017.1331538 PMC5496072

[52] Buzi RS , Weinman ML , Smith PB , Loudd G , Madanay FL . HIV stigma perceptions and sexual risk behaviors among black young women. J HIV/AIDS Soc Serv. 2018;17(1):69–85.

[53] Mugoya GC , Ernst K . Gender differences in HIV‐related stigma in Kenya. AIDS Care. 2014;26(2):206–213. 23795954 10.1080/09540121.2013.808733

[54] Hall KS , Manu A , Morhe E , Dalton VK , Challa S , Loll D , et al. Bad girl and unmet family planning need among sub‐Saharan African adolescents: the role of sexual and reproductive health stigma. Qual Res Med Healthc. 2018;2(1):55–64. 30556052 10.4081/qrmh.2018.7062 PMC6292434

[55] Calabrese SK , Tekeste M , Mayer KH , Magnus M , Krakower DS , Kershaw TS , et al. Considering stigma in the provision of HIV pre‐exposure prophylaxis: reflections from current prescribers. AIDS Patient Care STDs. 2019;33(2):79–88. 30715918 10.1089/apc.2018.0166 PMC6386080

[56] Raingruber B , Uwazie E , Bowie S . Women's voices: attitudes and behaviors of female Ghanaian sex workers regarding HIV prevention and AIDS‐related stigma. Issues Ment Health Nurs. 2010;31(8):514–519. 20624019 10.3109/01612841003646999

[57] Chan KY , Rungpueng A , Reidpath DD . AIDS and the stigma of sexual promiscuity: Thai nurses' risk perceptions of occupational exposure to HIV. Cult Health Sex. 2009;11(4):353–368. 19263260 10.1080/13691050802621161

[58] Gyan SE . Passing as “normal”: adolescent girls’ strategies for escaping stigma of premarital sex and childbearing in Ghana. SAGE Open. 2018;8(3). 10.1177/2158244018801421

[59] Hall KS , Morhe E , Manu A , Harris LH , Ela E , Loll D , et al. Factors associated with sexual and reproductive health stigma among adolescent girls in Ghana. PLoS One. 2018;13(4):e0195163. 29608595 10.1371/journal.pone.0195163 PMC5880390

[60] Castor C . Exploring HIV/AIDS and sexually transmitted infection risk, disease transmission knowledge, parental influence, and cultural stigma for young Haitian women via internet‐based focus groups and interviews. Teachers College, Columbia University; 2008.

[61] Mackworth‐Young CR , Bond V , Wringe A , Konayuma K , Clay S , Chiiya C , et al. “My mother told me that I should not”: a qualitative study exploring the restrictions placed on adolescent girls living with HIV in Zambia. J Int AIDS Soc. 2017;20(4):e25035. 29219248 10.1002/jia2.25035 PMC5810345

[62] Wong M , Myer L , Zerbe A , Phillips T , Petro G , Mellins CA , et al. Depression, alcohol use, and stigma in younger versus older HIV‐infected pregnant women initiating antiretroviral therapy in Cape Town, South Africa. Arch Womens Ment Health. 2017;20(1):149–159. 27815628 10.1007/s00737-016-0688-3 PMC5500299

[63] Ambaw F , Mossie A , Gobena T . Boy/girl friend and virginity values, and stigma related to condom among Jimma university students. Ethiop J Health Sci. 2010;20(3)169–177. 22434976 10.4314/ejhs.v20i3.69446 PMC3275841

[64] Nkwemu S , Jacobs CN , Mweemba O , Sharma A , Zulu JM . “They say that I have lost my integrity by breaking my virginity”: experiences of teen school going mothers in two schools in Lusaka Zambia. BMC Public Health. 2019;19(1):1–8. 30642304 10.1186/s12889-019-6394-0 PMC6332585

[65] Colombini M , Mutemwa R , Kivunaga J , Stackpool Moore L , Mayhew SH . Experiences of stigma among women living with HIV attending sexual and reproductive health services in Kenya: a qualitative study. BMC Health Serv Res. 2014;14(1):1–9. 25239309 10.1186/1472-6963-14-412 PMC4261560

[66] Boyes ME , Mason SJ , Cluver LD . Validation of a brief stigma‐by‐association scale for use with HIV/AIDS‐affected youth in South Africa. AIDS Care. 2013;25(2):215–222. 22774842 10.1080/09540121.2012.699668

[67] Clum G , Chung S‐E , Ellen JM , Interventions AMTNfHA . Mediators of HIV‐related stigma and risk behavior in HIV infected young women. AIDS Care. 2009;21(11):1455–1462. 20024724 10.1080/09540120902883069 PMC2860279

[68] Golub SA , Pena S , Fikslin R , Goldberg M , Radix A . Forecasted HIV stigma negatively impacts PrEP uptake: finding from a community‐based PrEP demonstration project. Ann Behav Med. 2018;21(11):1455‐1462. 10.1080/09540120902883069

[69] Harper GW , Lemos D , Hosek SG , Interventions AMTNfHA . Stigma reduction in adolescents and young adults newly diagnosed with HIV: findings from the project ACCEPT intervention. AIDS Patient Care STDs. 2014;28(10):543–554. 25216106 10.1089/apc.2013.0331 PMC4183905

[70] Nyblade L , Ndirangu JW , Speizer IS , Browne FA , Bonner CP , Minnis A , et al. Stigma in the health clinic and implications for PrEP access and use by adolescent girls and young women: conflicting perspectives in South Africa. BMC Public Health. 2022;22(1):1916. 36242000 10.1186/s12889-022-14236-z PMC9563466

[71] Skovdal M , Clausen CL , Magoge‐Mandizvidza P , Dzamatira F , Maswera R , Nyamwanza RP , et al. How gender norms and ‘good girl’ notions prevent adolescent girls and young women from engaging with PrEP: qualitative insights from Zimbabwe. BMC Womens Health. 2022;22(1):344. 35974360 10.1186/s12905-022-01928-2 PMC9379870

[72] Velloza J , Khoza N , Scorgie F , Chitukuta M , Mutero P , Mutiti K , et al. The influence of HIV‐related stigma on PrEP disclosure and adherence among adolescent girls and young women in HPTN 082: a qualitative study. J Int AIDS Soc. 2020;23(3):e25463. 32144874 10.1002/jia2.25463 PMC7060297

[73] Laher F , Salami T , Hornschuh S , Makhale LM , Khunwane M , Andrasik M , et al. PrEP stigma affects PrEP uptake: attitudes towards pre‐exposure prophylaxis (PrEP) amongst HIV vaccine trial participants in Soweto, South Africa. J Int AIDS Soc. 2021;24. 10.1002/jia2.25659

[74] Wechsberg WM , Browne FA , Ndirangu J , Bonner CP , Minnis AM , Nyblade L , et al. The PrEPARE Pretoria Project: protocol for a cluster‐randomized factorial‐design trial to prevent HIV with PrEP among adolescent girls and young women in Tshwane, South Africa. BMC Public Health. 2020;20(1):14. 32933510 10.1186/s12889-020-09458-y PMC7490774

[75] Gillespie D , Williams A , Wood F , Couzens Z , Jones A , Ma R , et al. Psychometric properties of an adapted stigma scale and experiences of stigma associated with HIV pre‐exposure prophylaxis use among men who have sex with men: a mixed methods study. AIDS Behav. 2023;27(7):2397–2410. 36622489 10.1007/s10461-022-03967-0 PMC10224859

[76] Budhwani H , Yigit I , Maragh‐Bass AC , Rainer CB , Claude K , Muessig KE , et al. Validation of HIV pre‐exposure prophylaxis (PrEP) medication scales with youth on PrEP: PrEP confidence scale and PrEP difficulties scale. AIDS Patient Care STDs. 2022;36(11):443–450. 36306520 10.1089/apc.2022.0072 PMC9700336

[77] Algarin AB , Hee Shrader C , Hackworth BT , Varas‐Diaz N , Fennie KP , Sheehan DM , et al. Development and validation of the community PrEP‐related stigma scale (community‐PSS). AIDS Educ Prev. 2021;33(2):120–128. 33821676 10.1521/aeap.2021.33.2.120 PMC8054770

[78] Siegler AJ , Wiatrek S , Mouhanna F , Amico KR , Dominguez K , Jones J , et al. Validation of the HIV pre‐exposure prophylaxis stigma scale: performance of Likert and semantic differential scale versions. AIDS Behav. 2020;24(9):2637–2649. 32157490 10.1007/s10461-020-02820-6 PMC7423865

[79] Powers JL , Tiffany JS . Engaging youth in participatory research and evaluation. J Public Health Manag Pract. 2006;12:S79–S87. 10.1097/00124784-200611001-00015 17035908

[80] Roberts ST , Mancuso N , Williams K , Nabunya HK , Mposula H , Mugocha C , et al. How a menu of adherence support strategies facilitated high adherence to HIV prevention products among adolescent girls and young women in sub‐Saharan Africa: a mixed methods analysis. J Int AIDS Soc. 2023;26(11):e26189. 37936551 10.1002/jia2.26189 PMC10630658

[81] Rousseau E , Katz AWK , O'Rourke S , Bekker LG , Delany‐Moretlwe S , Bukusi E , et al. Adolescent girls and young women's PrEP‐user journey during an implementation science study in South Africa and Kenya. PLoS One. 2021;16(10):e0258542. 34648589 10.1371/journal.pone.0258542 PMC8516266

[82] Roberts ST , Haberer J , Celum C , Mugo N , Ware NC , Cohen CR , et al. Intimate partner violence and adherence to HIV pre‐exposure prophylaxis (PrEP) in African women in HIV serodiscordant relationships: a prospective cohort study. J Acquir Immune Defic Syndr. 2016;73(3):313–322. 27243900 10.1097/QAI.0000000000001093 PMC5065369

[83] Camlin CS , Koss CA , Getahun M , Owino L , Itiakorit H , Akatukwasa C , et al. Understanding demand for PrEP and early experiences of PrEP use among young adults in rural Kenya and Uganda: a qualitative study. AIDS Behav. 2020;24:2149–2162. 31955361 10.1007/s10461-020-02780-x PMC7909847

[84] Lanham M , Wilcher R , Montgomery ET , Pool R , Schuler S , Lenzi R , et al. Engaging male partners in women's microbicide use: evidence from clinical trials and implications for future research and microbicide introduction. J Int AIDS Soc. 2014;17:19159. 25224618 10.7448/IAS.17.3.19159 PMC4163996

[85] Velloza J , Hosek S , Donnell D , Anderson PL , Chirenje M , Mgodi N , et al. Assessing longitudinal patterns of depressive symptoms and the influence of symptom trajectories on HIV pre‐exposure prophylaxis adherence among adolescent girls in the HPTN 082 randomized controlled trial. J Int AIDS Soc. 2021;24:e25731. 34164929 10.1002/jia2.25731 PMC8222844

[86] Katz AWK , Roberts S , Rousseau E , Khoza MN , Mogaka F , Bukusi E , et al. Qualitative analysis using social maps to explore young women's experiences with social support of their oral PrEP use in Kenya and South Africa. J Assoc Nurses AIDS Care. 2023;34(1):45–57. 36170124 10.1097/JNC.0000000000000363

[87] Stangl AL , Lloyd JK , Brady LM , Holland CE , Baral S . A systematic review of interventions to reduce HIV‐related stigma and discrimination from 2002 to 2013: how far have we come? J Int AIDS Soc. 2013;16(3S2):18734. 24242268 10.7448/IAS.16.3.18734 PMC3833106

[88] Roberts ST , Bann C , Hartmann M , Otticha S , Nyblade L , Marton T , et al. Developing a validated scale to measure PrEP stigma among adolescent girls and young women in western Kenya. 24th International AIDS Conference (AIDS 2022); 2022 29 July–2 Aug; Montreal, Canada.

[89] Turan JM , Elafros MA , Logie CH , Banik S , Turan B , Crockett KB , et al. Challenges and opportunities in examining and addressing intersectional stigma and health. BMC Med. 2019;17:1–15. 30764816 10.1186/s12916-018-1246-9 PMC6376691

[90] Crenshaw K . Race, gender, and sexual harassment. S Cal L Rev. 1991;65:1467.

[91] Dale SK , Ayala G , Logie CH , Bowleg L . Addressing HIV‐related intersectional stigma and discrimination to improve public health outcomes: an AJPH Supplement. Am J Public Health. 2022;112(S4):S335–S337. 35763724 10.2105/AJPH.2022.306738 PMC9241474

[92] Sievwright KM , Stangl AL , Nyblade L , Lippman SA , Logie CH , Maria Amélia de Sousa Mascena V , et al. An expanded definition of intersectional stigma for public health research and praxis. Am J Public Health. 2022;112(S4):S356–S361. 35763723 10.2105/AJPH.2022.306718 PMC9241457

[93] Stangl AL , Atkins K , Leddy AM , Sievwright KM , Sevelius JM , Lippman SA , et al. What do we know about interventions to reduce intersectional stigma and discrimination in the context of HIV? A systematic review. Stigma Health. 2023;8(3):393–408. 10.1037/sah0000414 PMC13095170 42016451

[94] Berger MT . Workable Sisterhood: The Political Journey of Stigmatized Women with HIV/AIDS, Princeton: Princeton University Pres; 2004. 10.1515/9781400826384

[95] Bond KT , Gunn A , Williams P , Leonard NR . Using an intersectional framework to understand the challenges of adopting pre‐exposure prophylaxis (PrEP) among young adult Black women. Sex Res Soc Policy. 2021;19(1):180–193. 10.1007/s13178-021-00533-6 PMC8992539 35401855

