# Supplementary material for: The development of a conceptual framework on PrEP stigma among adolescent girls and young women in sub‐Saharan Africa
Source: J Int AIDS Soc. 2024 Feb 20;27(2):e26213. doi: 10.1002/jia2.26213 (PMC10879468; doi:10.1002/jia2.26213)
Supplement: Supplementary file 1 — File S1: Literature review search strategy Information on file format. Document outlining the literature search terms used for all databases. [file JIA2-27-e26213-s001.docx]

**Supplementary material 1. Literature review search strategy**

| **Database** | **Search terms** |
| --- | --- |
| PubMed | #9 Add Search ("PrEP"[Title] OR "pre-exposure prophylaxis"[Title] OR Truvada[Title] OR (tenofovir[Title] AND emtricitabine[Title]) OR "human immunodeficiency virus"[Title] OR "preexposure prophylaxis"[Title] OR contraception[Title] OR contraceptive*[Title] OR condom*[Title] OR HIV*[Title] OR "sex"[Title] OR sexual[Title] OR sexuality[Title]) AND (stigma[Title] OR destigma*[Title] OR shame*[Title] OR shaming[Title] OR disgrace*[Title] OR ashamed[Title]) AND (adolescent*[Title] OR adolescence*[Title] OR teen*[Title] OR youth[Title] OR young[Title] OR "school age"[Title] OR high school*[Title] OR college*[Title] OR universit*[Title] OR sororit*[Title]) AND (female*[Title] OR girl*[Title] OR "women"[Title] OR sororit*[Title]) Filters: Publication date from 2009/01/01; English 17  #8 Add Search (("HIV"[Mesh] OR "Anti-HIV Agents"[Mesh] OR "HIV Infections"[Mesh] OR "human immunodeficiency virus"[Title] OR HIV*[Title]) AND ("Social Stigma"[Mesh] OR "Shame"[Mesh] OR stigma[Title] OR destigma*[Title] OR shame*[Title] OR shaming[Title] OR disgrace*[Title] OR ashamed[Title]) AND (((adolescent*[Title] OR adolescence*[Title] OR teen*[Title] OR youth[Title] OR young[Title]) AND (female*[Title] OR girl*[Title])) OR "young women"[Title])) Filters: Publication date from 2009/01/01; English 6  #5 Add Search (("Contraception"[Mesh] OR "Contraceptive Agents"[Mesh] OR "Sexuality"[Mesh] OR "Sexual Behavior"[Mesh] OR contraception[Title] OR contraceptive*[Title] OR condom*[Title] OR "sex"[Title] OR sexual[Title] OR sexuality[Title]) AND ("Social Stigma"[Majr] OR "Shame"[Majr] OR stigma[Title] OR destigma*[Title] OR shame*[Title] OR shaming[Title] OR disgrace*[Title] OR ashamed[Title]) AND ((("Adolescent"[Mesh] OR "Adolescent Health"[Mesh] OR "Young Adult"[Mesh] OR adolescent*[Title] OR adolescence*[Title] OR teen*[Title] OR youth[Title] OR young[Title]) AND ("Female"[Mesh] OR female*[Title] OR girl*[Title])) OR "young women"[Title]) NOT ("Adult"[Mesh] NOT ("Adolescent"[Mesh] OR "Adolescent Health"[Mesh] OR "Young Adult"[Mesh]))) Filters: Publication date from 2009/01/01; English 223  #2 Add Search (("Pre-Exposure Prophylaxis"[Mesh] OR "PrEP"[Title] OR "pre-exposure prophylaxis"[Title] OR Truvada[Title] OR (tenofovir[Title] AND emtricitabine[Title]) OR "preexposure prophylaxis"[Title]) AND ("Social Stigma"[Mesh] OR "Shame"[Mesh] OR stigma[Title] OR destigma*[Title] OR shame*[Title] OR shaming[Title] OR disgrace*[Title] OR ashamed[Title])) Filters: Publication date from 2009/01/01; English 68  #1 Add Search (("Pre-Exposure Prophylaxis"[Mesh] OR "HIV"[Mesh] OR "Anti-HIV Agents"[Mesh] OR "HIV Infections"[Mesh] OR "Contraception"[Mesh] OR "Contraceptive Agents"[Mesh] OR "Sexuality"[Mesh] OR "Sexual Behavior"[Mesh] OR "PrEP"[Title] OR "pre-exposure prophylaxis"[Title] OR Truvada[Title] OR (tenofovir[Title] AND emtricitabine[Title]) OR "human immunodeficiency virus"[Title] OR "preexposure prophylaxis"[Title] OR contraception[Title] OR contraceptive*[Title] OR condom*[Title] OR HIV*[Title] OR "sex"[Title] OR sexual[Title] OR sexuality[Title]) AND ("Social Stigma"[Mesh] OR "Shame"[Mesh] OR stigma[Title] OR destigma*[Title] OR shame*[Title] OR shaming[Title] OR disgrace*[Title] OR ashamed[Title]) AND ("Program Development"[Mesh] OR "Program Evaluation"[Mesh] OR intervention*[Title] OR program*[Title] OR evaluat*[Title]) AND ((("Adolescent"[Mesh] OR "Adolescent Health"[Mesh] OR "Young Adult"[Mesh] OR adolescent*[Title] OR adolescence*[Title] OR teen*[Title] OR youth[Title] OR young[Title]) AND ("Female"[Mesh] OR female*[Title] OR girl*[Title])) OR "young women"[Title])) Filters: Publication date from 2009/01/01; English 84 |
| Web of Science | #6 22 TITLE: (("PrEP" OR "pre-exposure prophylaxis" OR Truvada) AND (stigma OR destigma* OR shame* OR shaming OR disgrace* OR ashamed)) AND LANGUAGE: (English) Indexes=SCI-EXPANDED, SSCI, CPCI-S, CPCI-SSH Timespan=2009-2019  #5 4 TITLE: (("PrEP" OR "pre-exposure prophylaxis" OR Truvada) AND (stigma OR destigma* OR shame* OR shaming OR disgrace* OR ashamed)) AND TOPIC: ((adolescent* OR adolescence* OR teen* OR youth OR young OR "school age" OR "high school*" OR college* OR universit* OR sororit*) AND (female* OR girl* OR "women" OR sororit*)) AND LANGUAGE: (English) Indexes=SCI-EXPANDED, SSCI, CPCI-S, CPCI-SSH Timespan=2009-2019  #4 3 TOPIC: (("PrEP" OR "pre-exposure prophylaxis" OR Truvada) NEAR/4 (stigma OR destigma* OR shame* OR shaming OR disgrace* OR ashamed)) AND TOPIC: ((adolescent* OR adolescence* OR teen* OR youth OR young OR "school age" OR "high school*" OR college* OR universit* OR sororit*) NEAR/4 (female* OR girl* OR "women" OR sororit*)) AND LANGUAGE: (English) Indexes=SCI-EXPANDED, SSCI, CPCI-S, CPCI-SSH Timespan=2009-2019  #3 96 (#2 NOT #1) AND LANGUAGE: (English) Indexes=SCI-EXPANDED, SSCI, CPCI-S, CPCI-SSH Timespan=2009-2019  #2 119 TITLE: (("PrEP" OR "pre-exposure prophylaxis" OR Truvada OR (tenofovir AND emtricitabine) OR "human immunodeficiency virus" OR "preexposure prophylaxis" OR contraception OR contraceptive* OR condom* OR HIV* OR "sex" OR sexual OR sexuality) AND (stigma OR destigma* OR shame* OR shaming OR disgrace* OR ashamed)) AND TOPIC: ((adolescent* OR adolescence* OR teen* OR youth OR young OR "school age" OR "high school*" OR college* OR universit* OR sororit*) AND (female* OR girl* OR "women" OR sororit*)) AND LANGUAGE: (English) Indexes=SCI-EXPANDED, SSCI, CPCI-S, CPCI-SSH Timespan=2009-2019  #1 23 TITLE: (("PrEP" OR "pre-exposure prophylaxis" OR Truvada OR (tenofovir AND emtricitabine) OR "human immunodeficiency virus" OR "preexposure prophylaxis" OR contraception OR contraceptive* OR condom* OR HIV* OR "sex" OR sexual OR sexuality) AND (stigma OR destigma* OR shame* OR shaming OR disgrace* OR ashamed) AND (adolescent* OR adolescence* OR teen* OR youth OR young OR "school age" OR "high school*" OR college* OR universit* OR sororit*) AND (female* OR girl* OR "women" OR sororit*)) AND LANGUAGE: (English) Indexes=SCI-EXPANDED, SSCI, CPCI-S, CPCI-SSH Timespan=2009-2019 |
| PsycINFO | S4 TI ("PrEP" OR "pre-exposure prophylaxis" OR Truvada OR (tenofovir AND emtricitabine) OR "human immunodeficiency virus" OR "preexposure prophylaxis" OR contraception OR contraceptive* OR condom* OR HIV* OR "sex" OR sexual OR sexuality) AND (MM "Stigma" OR TI (stigma OR destigma* OR shame* OR shaming OR disgrace* OR ashamed)) AND (MM "Adolescent Attitudes" OR MM "Adolescent Behavior" OR MM "Adolescent Health" OR MM "Adolescent Mothers" OR MM "Adolescent Pregnancy" OR TI (adolescent* OR adolescence* OR teen* OR youth OR young OR "school age" OR "high school*" OR college* OR universit* OR sororit*)) AND (MM "Female Attitudes" OR TI (female* OR girl* OR "women" OR sororit*)) Limiters - Publication Year: 2009-2019; English 17  S3 TI (("PrEP" OR "pre-exposure prophylaxis" OR Truvada) AND (stigma OR destigma* OR shame* OR shaming OR disgrace* OR ashamed)) Limiters - Publication Year: 2009-2019; English 14  S2 TI (("PrEP" OR "pre-exposure prophylaxis" OR Truvada OR (tenofovir AND emtricitabine) OR "human immunodeficiency virus" OR "preexposure prophylaxis" OR contraception OR contraceptive* OR condom* OR HIV* OR "sex" OR sexual OR sexuality) AND (stigma OR destigma* OR shame* OR shaming OR disgrace* OR ashamed) AND (adolescent* OR adolescence* OR teen* OR youth OR young OR "school age" OR "high school*" OR college* OR universit* OR sororit*) AND (female* OR girl* OR "women" OR sororit*)) Limiters - Publication Year: 2009-2019; English 15  S1 (DE "HIV" OR DE "AIDS" OR DE "AIDS (Attitudes Toward)" OR DE "AIDS Prevention" OR DE "HIV Testing" OR DE "Sexuality" OR DE "Psychosexual Behavior" OR DE "Safe Sex" OR DE "Contraceptive Devices" OR DE "Condoms" OR DE "Diaphragms (Birth Control)" OR DE "Intrauterine Devices" OR DE "Oral Contraceptives" OR DE "Birth Control" OR DE "Contraceptive Devices" OR DE "Rhythm Method" OR TI ("PrEP" OR "pre-exposure prophylaxis" OR Truvada OR (tenofovir AND emtricitabine) OR "human immunodeficiency virus" OR "preexposure prophylaxis" OR contraception OR contraceptive* OR condom* OR HIV* OR "sex" OR sexual OR sexuality)) AND (DE "Prejudice" OR DE "Implicit Bias" OR DE "Stigma" OR DE "Shame" OR TI (stigma OR destigma* OR shame* OR shaming OR disgrace* OR ashamed)) AND (DE "Adolescent Attitudes" OR DE "Adolescent Behavior" OR DE "Adolescent Health" OR DE "Adolescent Mothers" OR DE "Adolescent Pregnancy" OR ZG "adolescence (13-17 yrs)" OR ZG "young adulthood (18-29 yrs)" OR TI (adolescent* OR adolescence* OR teen* OR youth OR young OR "school age" OR "high school*" OR college* OR universit* OR sororit*)) AND (DE "Female Attitudes" OR ZX "female" OR TI (female* OR girl* OR "women" OR sororit*)) Limiters - Publication Year: 2009-2019; English; Methodology: LITERATURE REVIEW, -Systematic Review 1 |
